# Impact of Vitamin D Levels on Progression-Free Survival and Response to Neoadjuvant Chemotherapy in Breast Cancer Patients: A Systematic Review and Meta-Analysis

**DOI:** 10.3390/cancers16244206

**Published:** 2024-12-17

**Authors:** Alessandro Ottaiano, Bianca Arianna Facchini, Marialucia Iacovino, Mariachiara Santorsola, Sergio Facchini, Giordana Di Mauro, Enrica Toscano, Monica Montopoli, Annabella Di Mauro, Vincenzo Quagliariello, Nicola Maurea, Gianluca Vanni, Alessia Bignucolo, Liliana Montella, Marco Materazzo, Mario Roselli, Oreste Claudio Buonomo, Massimiliano Berretta

**Affiliations:** 1 Istituto Nazionale Tumori di Napoli, IRCCS “G. Pascale”, Via M. Semmola, 80131 Naples, Italy; a.ottaiano@istitutotumori.na.it (A.O.); marialucia-iacovino@istitutotumori.na.it (M.I.); mariachiara.santorsola@istitutotumori.na.it (M.S.); 2 Division of Medical Oncology, Department of Precision Medicine, University of Campania Luigi Vanvitelli, 80138 Naples, Italy; biancaarianna.facchini@studenti.unicampania.it (B.A.F.); sergio.facchini@unicampania.it (S.F.); 3 School of Specialization in Medical Oncology, University of Messina, 98125 Messina, Italy; giordana.di.mauro@studenti.unime.it (G.D.M.); enrica.toscano@studenti.unime.it (E.T.); 4 Department of Pharmaceutical and Pharmacological Sciences, University of Padova, 35131 Padova, Italy; monica.montopoli@unipd.it; 5 Pathological Anatomy and Cytopathology Unit, Istituto Nazionale Tumori, IRCCS Fondazione G. Pascale, 80131 Naples, Italy; annabella.dimauro@istitutotumori.na.it; 6 Division of Cardiology, Istituto Nazionale Tumori-IRCSS-Fondazione G. Pascale, 80131 Naples, Italy; vincenzo.quagliariello@istitutotumori.na.it (V.Q.); n.maurea@istitutotumori.na.it (N.M.); 7 Breast Unit, Department of Surgical Science, PTV Policlinico Tor Vergata University, 00133 Rome, Italy; gianluca.vanni@ptvonline.it (G.V.); marco.materazzo@ptvonline.it (M.M.); o.buonomo@inwind.it (O.C.B.); 8 Department of Clinical and Experimental Medicine, University of Messina, Via Consolare Valeria, 98125 Messina, Italy; abignucolo@unime.it; 9 Division of Medical Oncology, “Santa Maria delle Grazie” Hospital, ASL Napoli 2 Nord, 80078 Pozzuoli, Italy; liliana.montella@aslnapoli2nord.it; 10 Medical Oncology Unit, Department of Systems Medicine, Tor Vergata University Hospital, 00133 Rome, Italy; mario.roselli@uniroma2.it

**Keywords:** breast cancer, neoadjuvant therapy, response, progression-free survival, meta-analysis, vitamin D

## Abstract

**Background:** Breast cancer remains the leading cause of cancer-related deaths among women despite advances in early detection. Neoadjuvant chemotherapy (NACT) is now standard for early-stage BC, with vitamin D (VD) emerging as a potential prognostic biomarker considering its positive pleiotropic effects. This review and meta-analysis assess the impact of baseline VD levels on outcomes in BC patients undergoing NACT. **Methods:** Inclusion criteria required patients to be over 18 years of age, have a pathologically confirmed BC diagnosis, and have their VD levels assessed prior to chemotherapy. Studies were included if they reported odds ratios (ORs) for response and/or hazard ratios (HRs) for PFS with 95% confidence intervals (CIs). A comprehensive literature search of PubMed/MEDLINE and Scopus/ELSEVIER (2014–2024) was conducted, and data were analyzed using fixed- and random-effects models, with Forest plots illustrating the results. Study quality and potential biases were assessed using the MINORS, NOS, and RoB2 scales, and statistical heterogeneity was evaluated with I^2^ statistics and funnel plots. **Results:** Six studies were included in the analysis. All studies addressed stages II and III, with three also including stage I. The meta-analysis covered data from 722 patients regarding NACT response and 1033 patients for PFS. The results revealed a 22% reduction in the likelihood of non-response to NACT associated with adequate VD levels (low/deficient VD vs. high/sufficient VD; OR: 0.78; 95% CI: 0.30–1.25; *p* = 0.001) and a 35% reduction in progression risk with sufficient baseline VD levels (low/deficient VD vs. high/sufficient VD; HR: 0.65; 95% CI: 0.33–0.97; *p* < 0.001). **Conclusions:** These findings highlight the significance of maintaining adequate vitamin D levels in BC treatment and encourage further studies to unravel the role of VD on cancer biology.

## Simple Summary

Among the pleiotropic functions of vitamin D, its involvement as an anticancer synergistic compound has recently emerged. The active form of VD has been shown to induce cell cycle arrest, cell apoptosis, and autophagy, and to suppress angiogenesis and metastatic progression in the TME via various signal transduction pathways. Interest has focused on the ability of VD to enhance the antitumor activity of some cancer drugs, suggesting its potential role as a chemosensitizer in breast cancer therapy. From our meta-analysis, it emerged that adequate baseline VD levels are associated with a 22% reduction in the risk of a non-response to NACT and a 35% reduction in the risk of disease progression. These results suggest a new potential role of VD as prognostic biomarker of PFS and therapeutic response.

## 1. Introduction

Breast cancer (BC) is the second most commonly diagnosed cancer in women globally, representing approximately 11.6% of all cancer cases in both sexes in 2022. Despite advances in early detection through widespread screening programs, particularly in developed nations, BC remains the leading cause of cancer-related death among women and the fourth leading cause of cancer-related death overall, resulting in over 665,000 deaths in 2022 [1].

BC is characterized by its significant histological heterogeneity, with the majority of cases classified as “no special type” (NST) and further categorized into Luminal A, Luminal B, HER2-enriched, and triple-negative BC (TNBC) based on the expression of estrogen receptor (ER), progesterone receptor (PR), Ki-67, and HER2 receptor [2]. This classification carries crucial prognostic and predictive implications, guiding treatment decisions [3]. The therapeutic landscape for BC has expanded with the development of increasingly effective drugs, including chemotherapy, hormone therapies (e.g., tamoxifen, anastrozole, letrozole, fulvestrant), anti-HER2 monoclonal antibodies (e.g., trastuzumab, pertuzumab), and, more recently, and anti-HER2 antibody–drug conjugates (ADCs) (e.g., trastuzumab–deruxtecan, trastuzumab–emtansine, datopotamab–deruxtecan, sacituzumab–govitecan), as well as immunotherapies (e.g., pembrolizumab, atezolizumab) [4].

Given the notable efficacy of these treatments in metastatic BC, some have been applied in earlier-stage settings as neoadjuvant therapies. These treatments have demonstrated significant success, particularly in TNBC and HER2-positive tumors, by enhancing response rates and improving time-to-event outcomes (progression and/or death) [5]. Neoadjuvant chemotherapy (NACT) has become the standard of care for most early-stage breast cancer cases, except for small tumors (T1a-T1b) without lymph node involvement, where surgery remains the preferred initial approach [6]. The primary goal of neoadjuvant therapy is to reduce tumor size or extent before surgery, thereby facilitating less invasive surgical options and enabling a more rapid and targeted approach to the early treatment of micrometastases. Concurrently, research into circulating biomarkers holds promise for further stratifying tumor biology and enhancing the efficacy of pre-surgical treatments. Among biomarkers, vitamin D has gained attention for its potential role in influencing the prognosis of BC patients in both metastatic [7,8] and non-metastatic settings [9,10]. Vitamin D3 (VD) is a fat-soluble molecule synthesized in the skin through ultraviolet light exposure and converted in the liver to 25-hydroxyvitamin D [25(OH)D], or calcidiol, by the enzyme 25-hydroxylase. Calcidiol is further converted in the kidneys to 1,25(OH)2D, or calcitriol, by the enzyme 1α-hydroxylase [11]. Calcitriol, due to its lipid-soluble nature, binds to specific cytoplasmic receptors, and after translocation to the nucleus, regulates the expression of genes involved in calcium homeostasis and bone metabolism [12]. Beyond these established functions, VD also impacts immune function, cardiovascular health, and cancer progression [13,14,15,16,17,18].

In this comprehensive review and meta-analysis, we aim to quantify the relationship between baseline VD levels prior to treatment and outcomes in BC patients undergoing neoadjuvant therapy.

## 2. Materials and Methods

This article presents a systematic review and meta-analysis exploring the relationship between pre-treatment circulating VD levels, specifically 25-hydroxyvitamin D [25(OH)D], and outcomes such as treatment response and progression-free survival (PFS) in BC patients undergoing NACT. Adhering to the 2020 PRISMA guidelines [19], this study was conducted based on a rigorously developed protocol, which was registered with PROSPERO (the Prospective Register of Systematic Reviews, managed by the National Institute for Health Research, London, UK) under ID580535. The protocol comprehensively details the selection criteria and methodologies employed throughout the review.

### 2.1. Search Strategy and Selection Criteria

A thorough manual search was conducted across two leading international databases, PubMed/MEDLINE and Scopus/ELSEVIER, to identify relevant studies on BC. This approach leveraged the advantages of a human-driven search strategy to ensure a detailed and context-aware inclusion process. Six researchers were divided into two independent teams for this task (Team 1: A.O., M.L.I., M.B.; Team 2: M.S., A.D.M., A.F.). Team 1 initiated the search, which was subsequently replicated by Team 2. This collaborative effort was designed to maximize the search’s sensitivity by incorporating the diverse expertise and perspectives of the team members. While this manual approach was time-consuming, it ensured a thorough understanding of the scientific context and a qualitative assessment of the studies that might otherwise have been overlooked by the automated systems. The databases and sources were reviewed independently by the different team members, who made comparisons at regular intervals to ensure the rigor of the selection. The search strategy utilized the keywords “breast cancer” AND “vitamin D” OR “cholecalciferol”. This search covered literature from January 2014 to 30 June 2024, and was conducted using PubMed/MEDLINE and Scopus/ELSEVIER, which are renowned for their comprehensive biomedical coverage. The selected time frame (2014–2024) was carefully chosen to ensure the inclusion of relevant and high-quality studies. This period reflects a consensus among the authors, considering the evolution of methodologies, improvements in study quality, and advancements in technology. By concentrating on these recent years, we capture the latest developments in neoadjuvant BC therapy while preserving a valuable historical perspective. This strategy strengthens the validity and applicability of our results, offering a comprehensive view of the predictive and prognostic roles of VD in this clinical context.

To identify relevant studies, we applied specific inclusion criteria. Only articles published in English were considered to reduce language and publication biases. We focused on studies involving individuals aged 18 and older with histologically confirmed BC. Eligible studies were required to report VD levels measured prior to the initiation of NACT. Additionally, the studies needed to provide odds ratios (ORs) and/or hazard ratios (HRs) with corresponding 95% confidence intervals (CIs) for treatment response and/or PFS, respectively. We examined all sections of the articles, including Supplementary Materials, to obtain the necessary OR and HR data.

**Figure 1:**
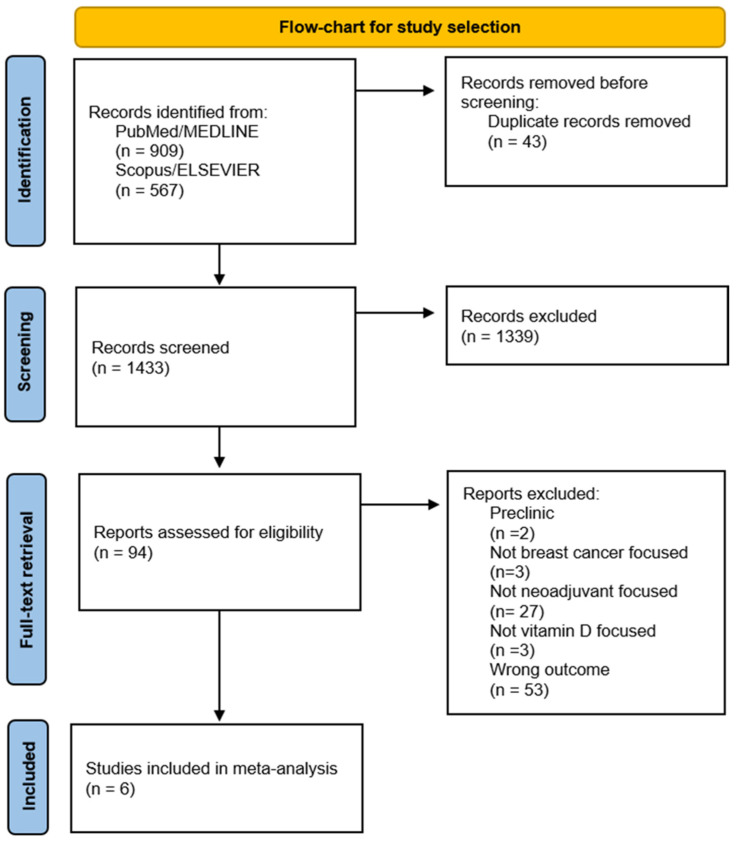
Flowchart of the study selection process.

The search included different types of VD supplementation, any study design, and the chemotherapy regimen used. Preclinical in vitro or animal studies were excluded from the initial analysis. For studies that examined VD in combination with other biomarkers, only those studies in which VD was analyzed independently were selected to minimize confounding factors in the interpretation of the actual data. This careful selection for the analysis of VD allows us to minimize bias as much as possible while deepening its actual role as a prognostic marker in BC. This method allowed us to improve the accuracy and reliability of the data. A detailed flowchart of the study selection process is provided in Figure 1.

### 2.2. Data Extraction

For each selected study, a range of information was extracted, including study size and design, the clinicopathologic characteristics of patients, methodology of VD analysis, follow-up, response and time-to-response data (including odds and hazard ratios), and 95% confidence interval (CI). Any disagreements or inconsistencies were discussed amongst the study team to reach a final agreement.

Primary objectives

This review and meta-analysis aim to evaluate two co-primary objectives in BC patients undergoing NACT:

- Assess the impact of VD levels on treatment response.
- Determine the influence of VD levels on PFS.

Quality assessment

Two working groups independently reviewed the results and methods of the selected studies to assess their potential bias and quality. The MINORS and NOS criteria were used to assess the non-randomized studies, while the RoB2 scale was used to assess the randomized studies [20,21,22] (see Supplementary Table S1). The two working groups then compared and discussed all discrepancies to reach a final consensus.

### 2.3. Statistical Methods

We conducted a meta-analysis to examine the association between pre-treatment VD levels and both response to neoadjuvant treatment and PFS in BC patients. The meta-analysis utilized both fixed-effect and random-effect models, applying the DerSimonian and Laird method [23]. In brief, the fixed-effect model assumes that the true effect size is consistent across all studies, with any variation in observed effect sizes attributed solely to random error. In contrast, the random-effect model allows for variability in the true effect size across studies, with observed differences reflecting both random error and true differences in effect sizes. Under the random-effect model, the true effect sizes are assumed to vary between studies, and the summary effect represents a weighted average of the individual study effects. This model often provides a more conservative estimate of the pooled hazard ratio (HR) and is preferred when heterogeneity is present across studies. The results are presented using Forest plots, which display odds ratios (ORs) and hazard ratios (HRs) with 95% confidence intervals (CIs) represented by error bars. The final pooled estimates of OR and HR are explicitly reported at the end of the graphs.

To enhance the clarity and consistency of result interpretation, it is important to explain that the OR represents the ratio of the odds of achieving a response to NACT between patients with low VD (LVD) levels and those with high VD (HVD) levels. An OR of 1.0 indicates no difference in the odds of a response between the two groups. An OR less than 1.0 suggests that patients with HVD have higher odds of a favorable response compared to those with LVD, implying a potential negative impact of LVD on treatment efficacy. The HR, on the other hand, compares the risk of disease progression (event probability) between patients with LVD and HVD during the observation period. A HR of 1.0 denotes equivalent risks between the two groups, while a HR greater than 1.0 indicates that LVD is associated with an increased risk of progression. If necessary, HRs will be recalculated using Altman’s method to ensure consistency in the comparison between the LVD and HVD groups [24].

Heterogeneity among the studies was evaluated using the I^2^ statistic [25]. The I^2^ statistic quantifies the percentage of variation across studies that can be attributed to real differences rather than random error. It is calculated as I^2^ = 100% × (Q − DF)/Q, where Q denotes Cochran’s heterogeneity statistic and DF represents the degrees of freedom. Negative I^2^ values are adjusted to zero, ensuring that I^2^ ranges from 0 to 100%. An I^2^ value of 0% signifies no observed heterogeneity, whereas higher values reflect increasing degrees of variability. Specifically, I^2^ values of 0–25% suggest minimal heterogeneity, indicating that differences among studies are likely due to chance. A values between 25 and 50% represents moderate heterogeneity, which may introduce some variability but generally does not critically affect the interpretation of results. An I^2^ of 50–75% indicates substantial heterogeneity, where significant variability among studies may impact the reliability of the meta-analysis, while values above 75% denote considerable heterogeneity, suggesting that the results of the meta-analysis should be interpreted with caution due to the high likelihood of variability influencing overall conclusions.

Potential publication bias was assessed using funnel plots [26]. This process involves several key steps. First, data from the selected studies are gathered, focusing on effect sizes such as odds ratios (ORs) or hazard ratios (HRs) along with their standard errors (SEs). These are then plotted on a scatter plot with effect sizes on the horizontal axis and precision (SEs) on the vertical axis. In the absence of bias, this plot should resemble a symmetrical funnel, where smaller studies are dispersed more widely, and larger studies are clustered towards the top. Any deviation from this expected funnel shape may suggest publication bias. Therefore, the interpretation of funnel plots requires careful examination of the symmetry and direction of any observed asymmetry. Irregularities might arise due to selective reporting or methodological differences. From a conceptual standpoint, the funnel represents the anticipated distribution of studies if no bias were present. Statistical tests such as Egger’s or Begg’s tests can be employed to formally detect and quantify asymmetry, providing a measurable indication of the potential presence of publication bias [26]. Identifying asymmetry helps to understand if positive or significant findings might disproportionately influence the overall results, with a symmetrical funnel plot suggesting a lower likelihood of publication bias and thereby supporting the internal validity of the study findings. Statistical analyses were conducted using MedCalc Statistical Software (MedCalc^®^ Statistical Software version 19.6, MedCalc Software Ltd., Ostend, Belgium) and Microsoft Excel^®^ for Windows, version 2302 (Microsoft Corporation, Redmond, WA, USA).

## 3. Results

Six studies that met the inclusion criteria were included in this meta-analysis [27,28,29,30,31,32]. The study selection process is illustrated in Figure 1. Table 1 provides a summary of the characteristics of the included articles. They vary in design, stages of cancer included, methods of VD assessment, and adjustment for seasonal variations in VD levels. Five of the six studies utilized a retrospective design, while one study was prospective (Charehbili, 2015). All studies included patients with stage II or III BC, with some also including stage I (Viala, 2018; Chiba, 2018; Tokunaga, 2022). The number of patients enrolled ranged from 82 to 374. VD assessment techniques differed across the studies. Radioimmunoassay (RIA) was the most employed method (Clark, 2014; Kim, 2018), often coupled with a Diasorin assay, while others used the enzyme-linked immunosorbent assay (ELISA) (Tokunaga, 2022) or the electrochemiluminescence binding assay (EBA) (Viala, 2018; Chiba, 2018). Only two studies (Kim, 2018; Charehbili, 2015) adjusted for seasonal variation in VD levels, a factor known to influence circulating levels of the biomarker. PFS was the primary outcome in four of the studies (Clark, 2014; Kim, 2018; Viala, 2018; Tokunaga, 2022), while overall survival (OS) was also included in one study (Viala, 2018). Response to NACT was frequently evaluated, with most studies categorizing patients based on pathological complete response (pCR) versus no-pCR. Only one study assessed responses using residual cancer burden (RCB), comparing RCB 0–1 (responders) versus RCB 2–3 (non-responders) (Clark, 2014). The comparisons of VD levels typically dichotomized patients into “deficient” and “sufficient” categories, with a threshold of 20 ng/mL, although one study (Charehbili, 2015) used the median value (50.99 nmol/L) for comparison. In addition to VD, several studies examined other biomarkers such as estrogen receptor (ER), progesterone receptor (PR), and HER-2 status, with Ki67 and tumor grade also being evaluated in some studies (Clark, 2014; Kim, 2018; Tokunaga, 2022). The quality of the studies, assessed using the MINORS, NOS, and RoB2 scales, is reported in the Supplementary Table S2.

**Figure 2:**
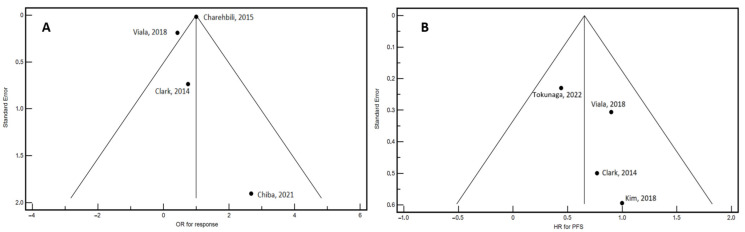
Funnel plots illustrating the selected studies for response to NACT (**A**) and progression-free survival (PFS) (**B**). The x-axis denotes the estimated outcome (OR: odds ratio; HR: hazard ratio), while the y-axis indicates the standard error. For response to NACT (**A**), the heterogeneity test yielded a significant result (Q = 9.9527, DF = 3, P = 0.0190), with an I^2^ (inconsistency) value of 69.86% (95% CI: 13.33% to 89.52%). Due to the considerable heterogeneity, a random-effects model was employed for the meta-analysis of the response to NACT. The funnel plot for PFS (**B**) shows no significant asymmetry, implying the absence of publication bias or small-study effects in this particular analysis [27,28,29,30,31,32].

A total of 722 estimates for response and 1033 estimates for PFS were included in the meta-analysis. To provide a descriptive overview relevant to treatment decisions and prognosis, Table 2 details the age, proportion of patients with positive estrogen and progesterone receptors, and response to NACT across the selected studies. The average ages were relatively consistent, except for Tokunaga’s (2022) study, which reported a notably older cohort with a mean age of 59 years. The number of patients with positive and negative hormone receptor status, as well as those achieving pathological complete response (pCR), varied among the studies. The NACT regimens used in each study are detailed in Supplementary Table S3. Significant heterogeneity in response to NACT was observed, as indicated by the asymmetrical distribution of studies in the funnel plot (Figure 2A). This variability necessitated the use of a random-effects model for analyzing NACT response. Conversely, the funnel plot for PFS showed no significant asymmetry, suggesting an absence of publication bias or small-study effects in this analysis (Figure 2B).

**Figure 3:**
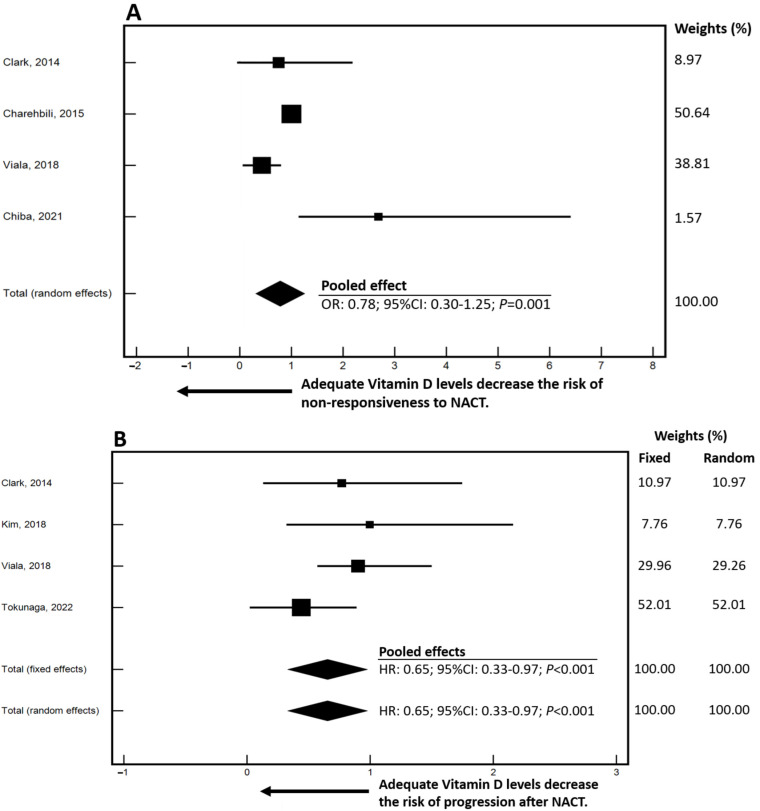
Forest graphs illustrating the pooled estimates of odds ratio (OR) of response (**A**) and progression-free survival (PFS) (**B**), stratified by varying levels of vitamin D in patients with breast cancer treated with neoadjuvant chemotherapy. The graphs present the pooled estimates using fixed- and/or random-effects models. To facilitate interpretation, an arrow on the x-axis indicates the trajectory of sufficient vitamin D levels and its relationship with varying risk, relative to the unit. Weights, which depend on the sample size and precision of estimates and reflect the relative contribution of each study to the meta-analysis, are reported to the right of the graphs. CI: Confidence Interval; HR: Hazard Ratio; NACT: NeoAdjuvant ChemoTherapy [27,28,29,30,31,32].

Table 3 provides a comprehensive summary of the estimates related to NACT (NACT) response and time-to-outcome as reported in the selected studies. This dataset, integral to the inclusion criteria, facilitates the computation of pooled estimates. It is important to note that most studies did not report median follow-up duration or median PFS. The impact of baseline VD levels on response and PFS was evaluated using odds ratios (OR) and hazard ratios (HR). Forest plots depicting the influence of VD on OR and PFS are presented in Figure 3A,B, respectively. The pooled data analysis revealed a 22% decrease in the risk of non-response to NACT associated with adequate baseline VD levels, based on data from 722 patients and analyzed using random-effects models (HR: 0.78, 95% CI: 0.30–1.25; *p* = 0.001). In terms of progression risk, the analysis of 1033 patients showed a 35% reduction in the risk of progression, with consistent findings across both fixed- and random-effects models (HR: 0.65; 95% CI: 0.33–0.97; *p* < 0.001). These results indicate that maintaining adequate 25-hydroxyvitamin D levels prior to starting NACT in BC patients significantly enhances treatment response and improves PFS.

## 4. Discussion

NACT has become a key and widely endorsed approach in BC treatment, according to leading international guidelines [6]. The expanding evidence highlighting the significant role of VD in influencing BC biology emphasizes the importance of examining baseline VD levels in patients undergoing this treatment. Considering the fragmented and uncertain current literature, coupled with the absence of comprehensive studies on the topic, we deemed it both scientifically justified and valuable to undertake a meta-analysis. Our goal was to determine the extent of the effect of VD in this context and to assess whether this influence was significant. To the best of our knowledge, this is the first study that specifically reports the effect of baseline VD levels in this BC setting. The present meta-analysis reveals that adequate baseline VD levels are associated with a 22% reduction in the risk of non-response to NACT and a 35% reduction in the risk of disease progression. These findings highlight the significant role of adequate levels of VD in enhancing both NACT response and PFS in BC patients.

Recent meta-analyses in the literature have correlated a high prevalence of VD insufficiency in patients with newly diagnosed BC [7,8] or have investigated the effect of VD levels on survival [9], regardless of the stage or clinical–therapeutic setting, hypothesizing a pathophysiological link to BC development or progression.

In recent years, a growing body of evidence has highlighted the effects of VD on BC cells, which are worth discussing, although not exhaustively. VD plays key roles in BC biology, among which is the inhibition of cancer cell proliferation. VD inhibits BC cell growth by inducing cell cycle arrest in the G1 phase, thereby preventing progression to DNA replication and mitosis. This mechanism is supported by studies showing that calcitriol treatment reduces the expression of cyclins and cyclin-dependent kinases, crucial for cell cycle progression [33]. Beyond its antiproliferative properties, VD promotes apoptosis, or programmed cell death, in BC cells. This effect is mediated through the upregulation of pro-apoptotic genes such as BAX and the downregulation of anti-apoptotic genes like BCL-2 [34,35]. Furthermore, VD exerts significant effects on the migration and invasiveness of BC cells by modulating the epithelial-to-mesenchymal transition (EMT), a process integral to cancer metastasis. EMT involves the loss of epithelial cell adhesion and the acquisition of migratory and invasive traits. VD counteracts EMT by upregulating E-cadherin, a protein essential for maintaining epithelial cell adhesion, while downregulating N-cadherin and vimentin, which are markers of mesenchymal transition [36]. This regulatory effect ultimately reduces the metastatic potential of BC cells. Moreover, VD influences matrix metalloproteinases (MMPs), key enzymes in extracellular matrix degradation and cancer cell invasion. It specifically downregulates the expression of MMP-2 and MMP-9, thereby limiting the invasive potential of BC cells [37]. Additionally, it exerts beneficial effects on BC-associated fibroblasts by reducing their ability to promote cancer cell proliferation and migration within the tumor microenvironment [38]. The immune-modulatory role of VD is particularly pertinent in BC. It enhances anti-tumor immune responses by influencing various immune cells, including promoting the differentiation of monocytes into macrophages with anti-tumor properties and improving the capacity of dendritic cells to activate T cells [39]. This immune activation facilitates the recognition and elimination of cancer cells [40]. Furthermore, VD helps reduce chronic inflammation, which is implicated in cancer progression and chemotherapy resistance. By modulating cytokine production, such as increasing levels of anti-inflammatory cytokines like IL-10 and decreasing pro-inflammatory cytokines like IL-6, VD contributes to a more favorable immune environment against BC [41].

Anthracyclines and taxanes are the most commonly anticancer drugs in NACT regimens. Interest has focused on the ability of VD to enhance the antitumor activity of anthracyclines, suggesting its potential role as a chemosensitizer in breast cancer therapy. The mechanisms by which VD and/or analogs enhance the cytotoxic activity of anticancer drugs have been extensively studied. It is well known that the anticancer effect of anthracyclines occurs via the inhibition of topoisomerase II and the ROS-mediated cytotoxicity that follows the generation of superoxide [42]. Ravid and colleagues found that pretreatment of MCF-7 breast cancer cells with VD significantly reduces both the expression and activity of Cu/Zn superoxide dismutase (Cu/Zn SOD), suggesting an increased sensitivity of cancer cells to ROS species [43,44]. Despite their widespread use, anthracyclines exhibit cardiotoxicity, which is a limiting side effect and sometimes leads to heart failure via endothelial cell death. In general, damage to endothelial cells by anthracyclines occurs via oxidative stress and the resulting inflammation [45]. It is known that endothelial cells can express vitamin D receptors (VDR) and produce VD, suggesting a potential regulatory function of these cells and a cardioprotective role of VD during anthracycline therapy. The study by Chen et al. showed that VD protects human aortic endothelial cells (HAECs) from doxorubicin damage. The researchers proposed a mechanism of action based on the upregulation of IL-10 expression, a cytokine involved in both downregulating the transcription of inflammatory cytokines and regulating the AMPKα/SIRT1/FOXO3a signaling pathway, which is normally inhibited by doxorubicin [46]. Less is known about the role of VD and taxanes’ efficacy. However, some studies highlighted the correlation of pre-treatment VD deficiency and the onset of severe peripheral neuropathy probably due to a mechanical hypersensitivity induced by microglial activation [47,48]. VD supplementation can improve this condition via nerve growth factor (NGF) stimulation and pro-inflammatory cytokine inhibition. An in vitro study of the effect of VD on p53-positive triple-negative breast cancer cells highlighted its synergistic effect with paclitaxel. VD enhanced the antitumor activity of paclitaxel, specifically reducing the drug IC_50_ measurement [49].

A previous meta-analysis that included all stages of breast cancer demonstrated a significant linear dose–response relationship between circulating 25-OH-D levels and overall survival [9]. This finding aligns with the positive effects of VD not only in the neoadjuvant setting but also in advanced disease stages. A key distinction between the present study and the meta-analysis by Kejia et al. [9] is that the current analysis specifically includes studies measuring VD levels prior to the initiation of therapy, thereby capturing the baseline biochemical status of endogenous VD.

Some limits of our study deserve to be revealed and discussed. Despite the inclusion of studies with large sample sizes, and good-quality scores, methodological heterogeneity was observed. Most of the included studies (five out of six) employed a retrospective design. Retrospective studies are inherently limited by their reliance on existing data, which can introduce biases related to data quality and completeness and are more susceptible to selection bias and confounding variables that may not be adequately controlled. The studies included in this meta-analysis employed different methods to assess VD levels, including RIA, ELISA, and EBA. Different methods have different sensitivities and specificities, which could impact the classification of VD status as deficient or sufficient and influence the study outcomes. Standardizing VD assessment methods or accounting for these variations in the analysis could help address this issue and improve the robustness of the conclusions drawn. Furthermore, only two studies (Kim, 2018; Charehbili, 2015) adjusted for seasonal variation in VD levels. Seasonal fluctuations can significantly affect circulating VD levels due to variations in sunlight exposure and dietary intake. The lack of adjustment for these variations in most studies introduces potential confounding, which could skew the results. Another notable limitation is the lack of reporting on median follow-up duration and median PFS in most studies. The duration of follow-up is crucial for understanding long-term outcomes and the stability of observed effects. The absence of detailed follow-up data may obscure the true impact of baseline VD levels on long-term outcomes.

## 5. Conclusions

VD exhibits well-established anti-carcinogenic effects in BC and has the potential to modulate immune responses, potentially augmenting the effectiveness of NACT. Our meta-analysis underscores the need for future research to explore the potential of VD supplementation as a novel strategy to enhance clinical outcomes in this context.

## Figures and Tables

**Table 1:** Characteristics of the selected studies.

First Author, Year	Study Design	Stage	Vitamin D Assessment	VD Adjusted per Season	N. of Patients	TTO	Response to NACT	Comparison Modalities *	Other Biomarkers Evaluated
Clark, 2014 [27]	R	II, III	RIA	No	82	PFS	Yes (RCB 0–1) vs. No (RCB 2–3)	Deficient: <20 ng/mL Sufficient: ≥20 ng/mL	Ki67, Bcl2 and tumor grade
Charehbili, 2015 [28]	P	II, III	NA	Yes	169	NA	pCR vs. no-pCR	Low: vitamin D < the median of measured values (50.99 nmol/L) High: vitamin D > 50.99 nmol/L	ER, PR
Kim, 2018 [29]	R	II, III	RIA	Yes	374	PFS	pCR vs. no-pCR	Deficient: <20 ng/mL Sufficient: ≥20 ng/mL	ER, PR, HER-2, Ki67
Viala, 2018 [30]	R	I, II, III	EBA, RIA	No	327	PFS, OS	pCR vs. no-pCR	Deficient: <20 ng/mL Sufficient: ≥20 ng/mL	ER, PR, HER-2
Chiba, 2018 [31]	R	I, II, III	EBA, RIA	No	144	NA	pCR vs. no-pCR	Deficient: <20 ng/mL Sufficient: ≥20 ng/mL	ER, PR, HER-2
Tokunaga, 2022 [32]	R	I, II, III	ELISA	No	250	PFS	pCR vs. no-pCR	Deficient: <20 ng/mL Sufficient: ≥20 ng/mL	ER, PR, HER-2, Ki67

* All studies included baseline assessments of Vitamin D. EBA electrochemiluminescence binding assay; ELISA: Enzyme-Linked Immunosorbent Assay; ER: estrogen receptor; NA: not assessed; NACT: neoadjuvant chemotherapy; OS overall survival; P: prospective; PFS: progression free survival; pCR: pathological complete response; PR: progesterone receptor; R: retrospective; RCB: residual cancer burden; RIA: Radioimmunoassay; TTO: time-to-outcome.

**Table 2:** Clinico-pathological characteristics of patients included in the selected articles.

Author	Year	Age (Mean)	Hormone Receptor		Response to NACT	
			**Positive, *n***	**Negative, *n***	**pCR, *n***	**No pCR, *n***
Clark [27]	2014	48.1	50	32	12	70
Charehbili [28]	2015	48.0 *	142	27	NR **	NR **
Kim [29]	2018	48.7	240	134	97	277
Viala [30]	2018	50	143	184	107	220
Chiba [31]	2018	NA	82	62	48	96
Tokunaga [32]	2022	59 *	178	72	60	190

* Median instead of mean age. ** The data are available in a prior publication by the authors; however, they are not explicitly provided in the selected article, which includes a different number of patients with available vitamin D measurements from the NEOZOTAC trial (BOOG 10-01). NR: not reported; NACT: neoadjuvant chemotherapy; pCR: pathological complete response.

**Table 3:** Detailed risk of recurrence and pathological response to NACT in the selected articles according to vitamin D levels.

Author	Year	Median Follow-Up (Months)	Median PFS (Months)	Response to NACT			PFS		
				HR	CI	*P*	HR	CI	*P*
Clark [27]	2014	NA	NA	0.75	0.14–2.19	0.535	0.77	0.34–1.75	0.535
Charehbili [28]	2015	NA	NA	1.00	0.97–1.03	0.76	NA	NA	NA
Kim [29]	2018	52.3 *	NA	NA	NA	0.795	0.998	0.461–2.163	0.997
Viala [30]	2018	63.6	NR	0.43	0.2–0.8	0.01	0.9	0.6–1.5	0.8
Chiba [31]	2018	NA	NA	2.68	1.12–6.41	0.03	NA	NA	NA
Tokunaga [32]	2022	NA	NA	NA	NA	0.4133	2.28	1.12–5.03	0.0231

* Mean instead of median; CI: confidence interval; HR: hazard ratio; PFS: progression-free survival; NA: not assessed; NR: not reached; *P*: *p*-value.

## Data Availability

No new data were created or analyzed in this study. Data sharing is not applicable to this article.

## Supplementary Materials

The following supporting information can be downloaded at: https://www.mdpi.com/article/10.3390/cancers16244206/s1, Table S1: Methodological aspects and outputs of the quality assessment scales employed in the study. Table S2: Study quality assessment. Table S3: Detailed regimens of NACT (neoadjuvant chemotherapy) used in the selected articles.
