# Supplementary material for: Impact of Vitamin D Levels on Progression-Free Survival and Response to Neoadjuvant Chemotherapy in Breast Cancer Patients: A Systematic Review and Meta-Analysis
Source: Cancers (Basel). 2024 Dec 17;16(24):4206. doi: 10.3390/cancers16244206 (PMC11674590; doi:10.3390/cancers16244206)
Supplement: Supplementary file 1 [file cancers-16-04206-s001.zip › cancers-3345479-supplementary.pdf]

Supplementary Table S1. Methodological aspects and outputs of the quality assessment scales employed**.**

**Aspect evaluated**	**MINORS**	**NOS**	**RoB2**
Study design	Explicit criteria for assessing non-randomized studies	Differentiates between cohort and case-control studies	Appraises the randomization process and study design
Control group	Presence and appropriateness of a control group	Assesses the selection and comparability of the control group	Evaluates allocation concealment, blinding, and baseline similarities
Baseline comparability	Evaluates baseline comparability between groups	Examines comparability based on characteristics and confounders	Assesses baseline similarities and adjustments
Patient selection	Clear description of patient selection process	Criteria for patient selection and representativeness of the sample	Evaluates the recruitment process and eligibility criteria
Endpoint assessment	Adequate reporting and evaluation of study endpoints	Assess the outcome measurement and ascertainment of outcomes	Evaluates outcome measurement and reporting
Statistical analysis	Considers statistical analysis and power calculation	Considers statistical methods, appropriateness, and power	Examines statistical analysis, bias, and precision
Loss to follow-Up	Addresses and accounts for loss to follow-up	Considers loss to follow-up and adequacy of follow-up duration	Evaluates completeness of follow-up and handling of missing data
Confounding factors	Assesses the control of confounding factors	Addresses confounding factors and control measures	Examines methods to control confounding and other biases
Interventions/Exposures	Evaluates the description and appropriateness	Examines exposure/intervention definition and measurement	Assesses intervention/exposure, randomization, and blinding
Quality of reporting	Considers the overall quality of reporting	Examines the clarity and completeness of reporting	Assesses reporting bias and selective outcome reporting
Score range (Min - Max)	0 - 16	0 - 9 (for cohort or case-control studies), 0 - 10 (for cohort studies)	0 - 11
Main aspect described	Assesses the overall methodological quality of non-randomized studies	Focuses on selection, comparability, and outcome assessment for observational studies	Evaluates the risk of bias related to study design

Supplementary Table S2. Study quality assessment.

First author, year	Study design	MINORS score	NOS score	RoB2 assessment
Clark, 2014	R	13	7	NA
Charehbili, 2015	P	NA	NA	Some concerns
Kim, 2018	R	11	8	NA
Viala, 2018	R	10	7	NA
Chiba, 2018	R	10	7	NA
Tokunaga, 2022	R	9	7	NA

MINORS: Methodological Index for Non-Randomized Studies; NA: not applicable; NOS: Newcastle–Ottawa Scale; P: Prospective; R: Retrospective; RoB2: Risk-of-Bias v.2.

Supplementary Table S3. Detailed regimens of NACT (neoadjuvant chemotherapy) used in the selected articles.

Author	Year	Regimens of NACT
Clark	2014	Anthracycline and taxanes. Women with HER2+ tumors were excluded.
Charehbili	2015	Docetaxel 75 mg/m^2, adriamycin 50 mg/m^2 and cyclophosphamide 500 mg/m^2, with or without zolendronic acid (4 mg i.v. within 24 h after infusion of chemotherapy).
Kim	2018	Four cycles of anthracycline plus cyclophosphamide (AC) followed by four cycles of taxane ± titanium silicate-1, or - AC alone, or cyclophosphamide, methotrexate, and fluorouracil, or taxane, carboplatin, and bevacizumab, or anthracycline plus taxane or taxane, anthracycline, and cyclophosphamide, or taxane plus trastuzumab. Adjuvant endocrine therapy was initiated according to the hormone receptor status.
Viala	2018	Sequential anthracycline and/or taxane-based chemotherapy, with the adjunction of HER2-directed therapies for HER2+ tumors (6 to 8 cycles). Adjuvant endocrine therapy was initiated according to the hormone receptor status. Women with HER2+ tumors received the recommendation for adjuvant trastuzumab per standard of care guidelines.
Chiba	2018	Sequential anthracycline and/or taxane-based chemotherapy, with the adjunction of trastuzuamb at the beginning of first taxane cycle for HER2+ tumors.
Tokunaga	2022	Standard regimens of anthracyclines and/or taxanes with the adjunction of trastuzumab for HER2+ tumors.
